# The relationship between community workers’ occupational stress, social support and psychological health: an empirical study in Liaoning Province, China

**DOI:** 10.3389/fpsyg.2024.1305432

**Published:** 2024-10-03

**Authors:** Qin Sen, Zhang Lei

**Affiliations:** School of Humanities and Law, Northeastern University, Shenyang, China

**Keywords:** community workers, psychological health, occupational stress, social support, moderating effect

## Abstract

**Background:**

With increasing urbanization in China, the community has assumed the important task of social governance and service provision, which has resulted in an increase in community workers’ occupational stress. To date, researchers have studied ways to improve community workers’ working ability, but have paid less attention to their health, especially their psychological health. This study examined the relationship between occupational stress, social support and community workers’ psychological health.

**Methods:**

A total of 545 community workers from 14 cities in Liaoning Province completed a questionnaire including the Occupational Adaptability Scale for Employees (OASE), the Social Support Rating Scale (SSRS) and the SCL-90-R scale (a measure of psychological health). Correlational and regression analyses were conducted.

**Results:**

Community workers’ occupational stress mainly derives from their workload, career development opportunities and performance appraisals. Participant’s psychological health scores were significantly negatively correlated with occupational stress and significantly positively correlated with social support. Social support plays a moderating effect in the relationship between community workers’ occupational stress and their psychological health.

**Conclusion:**

It is necessary to establish an effective community support system, reduce the burden on the community, optimize existing work processes and performance appraisal system, create a positive working environment and organizational culture, and promote employee’s psychological health.

## 1 Introduction

With increasing urbanization and modernization, communities have become the basic unit of social governance and public service provision in China. Community workers provide a direct link between residents and service providers, maintain the closest contact with them and safeguard their basic rights and interests. Community workers play an important role, especially in COVID-19 prevention. However, the development of communities is constrained by resources such as manpower, financial resources, and power in China. Community workers undertake complex tasks, are required to respond to the diverse needs of residents to resolve conflicts in the community, face enormous pressure and challenges, and are prone to psychological health problems such as anxiety and depression (Clifford et al., 2014). Empirical experience indicates that if psychological health issues are not effectively addressed, a person’s ability to work will be negatively impacted and service efficiency will be reduced (Song et al., 2021; Zhang et al., 2019). According to previous survey findings, the proportion of Chinese community workers with various psychological problems reached more than 60%, which is twice that of the general population (Asante et al., 2019). This shows that the psychological health of community workers is an important issue that cannot be ignored, but the issue has not attracted widespread attention from society. In particular, there is limited discussion and research on the causes of psychological health problems in this population.

Social support is a cross-concept of psychology and sociology, which refers to the various types of support that individuals receive from the social system (Cohen and Wills, 1985), including both objective support at the material level (funds, resources, skills, etc.) and subjective support at the emotional level (understanding, respect, praise, etc.). Adequate support protects the individual from the adverse effects of external pressures and enhances interpersonal communication and social connections (Naslund et al., 2016). The relevant theories of social support emphasize that in a complex social system, an individual cannot exist apart from the whole and must integrate into social networks to achieve the necessary sense of belonging and security, as well as positive self-worth. Especially in crisis situations, social support can enhance an individual’s ability to evaluate and manage his/her affairs, reduce the negative impact of external pressure on the individual, and provide a positive emotional experience (Cobb, 1976).

Community workers have worked in stressful environments for a long time, and their need for social support is self-evident. At present, national policy clearly calls for increasing resource support for community construction, increasing care for community workers and reducing their work pressure. In view of this, this study conducted a survey in 14 cities in Liaoning Province, using questionnaires to measure occupational stress, social support, and the psychological health of community workers. Moderating effects analysis was used to investigate the moderating effect of social support on occupational stress and psychological health, hoping that the results will provide data to inform improvements in community workers’ health and improve community construction systems.

## 2 Literature review

### 2.1 Community workers

The definition of community workers is divided into both a broad and narrow sense. The broad sense generally refers to full-time and part-time personnel engaged in governance and service within the community, including community party committee members and neighborhood committee personnel, NGO practitioners, and community volunteers, etc. The narrow sense refers to community party committee members and neighborhood committee personnel (Palmer et al., 2011). To enhance the rigor and reliability and reduce the impact of potentially confounding factors in the research, the narrow definition was adopted in this study.

Previous studies on community workers have primarily focused on how to improve professionalism (Huang et al., 2018), optimize personnel structure (Wang et al., 2023), reduce work burden (Wable et al., 2022), and improve work performance (Li et al., 2024), etc. The lack of investigation and discussion of their health, especially psychological health, has resulted in a limitation of research perspectives.

### 2.2 Social support

Research on social support originated in the 1960s. Early researchers regarded social support as social ties and generally believed that social support could help individuals cope with difficulties in life and work (Baumeister and Leary, 1995). Later scholars realized that different types of social relationships can provide a range of social supports, which influence people differently. Tough et al. (2017) indicated that the social support that communities can provide for individuals includes four types: attribution support, self-esteem satisfaction support, material support and approval support. Park et al. (2021) indicated the complexity of social support and divided it into objective support and subjective support. The former refers to material and visible support, including materials, money, services, companionship and information while the latter refers to the emotional support that individuals experience, including respect, praise, and understanding in society.

Scholars have also focused on the mechanism by which social support functions and proposed three hypotheses – the main effect model, the buffering effect model, and the dynamic model. The main effect model holds that social support has a general gain effect, which inevitably leads to the improvement of an individual’s health status (Leahy-Warren et al., 2012). The buffering effect model states that social support can eliminate external stress and thus have a positive impact on an individual’s health status (Weil, 2015). The dynamic model holds that social support and an individual’s health are not simple positive influences, but a complex interactive relationship, and change according to changes in time and circumstances (Laquidara and Lincoln, 2024).

Based on the aforementioned research findings, scholars have gradually recognized the significant role of social support in individual health and organizational development. Katherine et al. (2010) empirically tested that organizations can improve employee health conditions, reduce operational costs, and enhance productivity by improving the work environment and implementing health programs. Yilmaz et al. (2018) found that employees’ perceived social support can affect their sense of meaning, thereby enhancing their creativity and proactivity. Isidro et al. (2018) discovered that employees’ perceived social support affects the development performance of their organizations through the mediating variable of job satisfaction.

### 2.3 Definition and factors that influence psychological health

According to the World Health Organization (WHO), psychological health includes three aspects: a positive personality, sound ability to deal with issues, and positive interpersonal relationships. Scholars define psychological health from different perspectives and criteria. For example, Liu and Stewart (2024) believes that psychological health means that individuals have positive vitality, good internal experiences, strong social adaptability and are able to control their emotions in the face of external pressures, to achieve their potential and function socially. Williams (2016) considers that psychological health refers to a sound state of internal and external adjustment, including not only a sense of stability of their internal environment, but also adaptability to the external environment.

Existing studies have shown that the factors affecting psychological health are multifaceted and differ in different occupational groups. Lin (2022) conducted a survey on couriers and found that the factors affecting their psychological health mainly came from occupational pressure with greater pressure corresponding to lower health indexes. McIntyre et al. (2019) found that factors such as income, working hours, rational division of labor, and career promotion significantly affected teacher’s psychological health. Lan et al. (2014) found that work stress, leadership skills, colleague’s trust, interpersonal relationships, and salary affected the degree of job burnout, and healthcare workers’ psychological health in a survey study.

The above research findings have clarified the core concepts relevant to this study and have touched upon the possible interplay between occupational stress, social support, and psychological health. However, the occupation of community workers has unique characteristics, and it is uncertain whether previous research findings are applicable to their specific context. This issue requires further exploration and verification, which provides an opportunity for this study to delve deeper into the subject.

## 3 Methods

### 3.1 Sample data sources

The data for this study were derived from a survey conducted by the Urban and Rural Community Construction Research Institute of Northeastern University from 2021 to 2023, aimed at exploring the level of community development in Liaoning Province. This research constitutes a sub-project of the said initiative. The reasons for selecting Liaoning Province as the site for this study are primarily as follows: First, Liaoning Province has a solid foundation in economic and social development, with a considerable number and variety of communities. Second, Liaoning Province was among the early adopters in advancing community construction initiatives, enacting a series of policy documents aimed at community workers, which has led to the formation of a relatively comprehensive system for community management, support, and development. The occupational status of these workers is representative of the general situation in China. Therefore, conducting research on community workers in Liaoning Province is both typical and representative.

**Figure 1:**
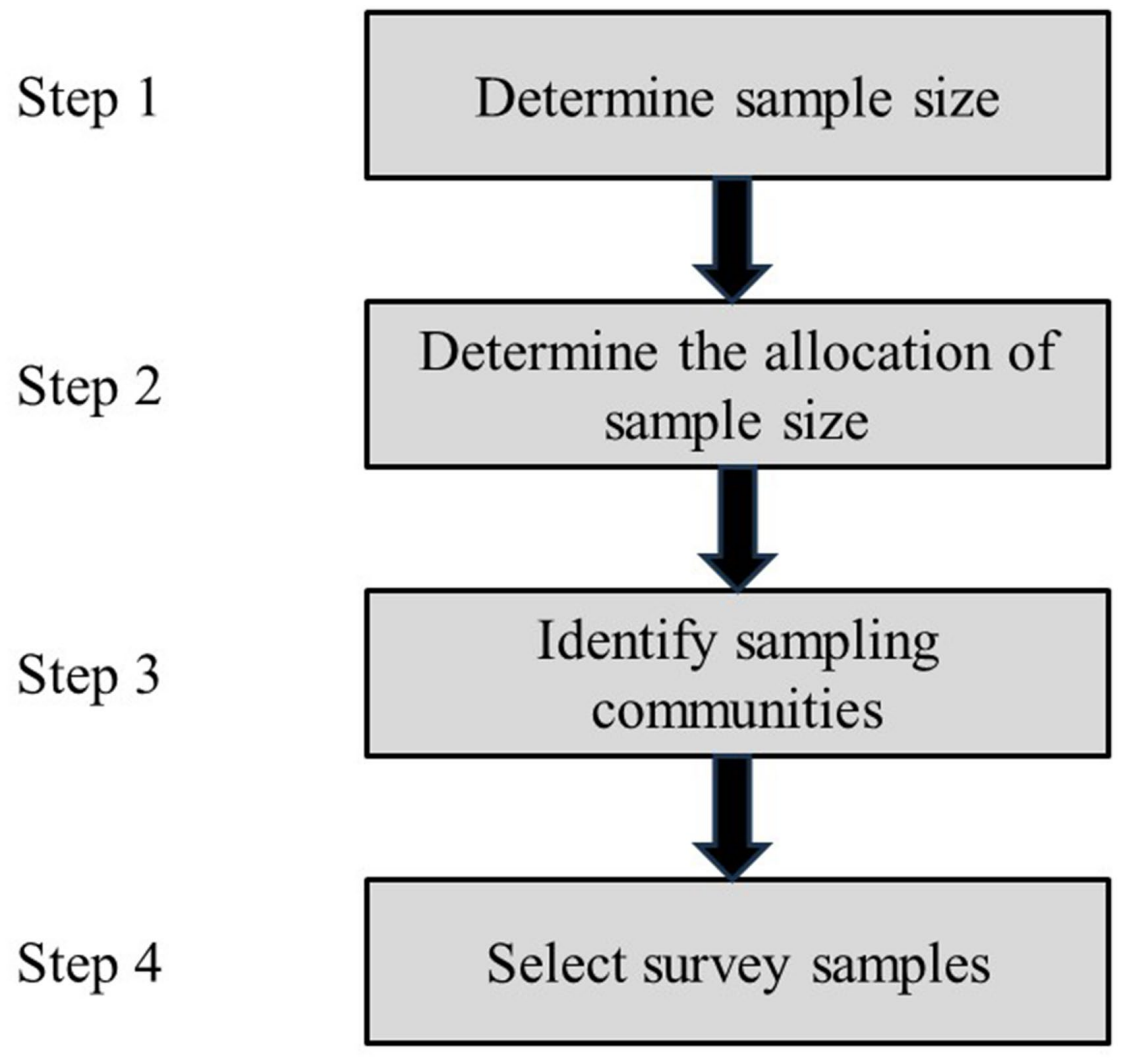
Chart of sample selection.

This study adopted a multi-stage stratified cluster sampling method for the survey. Step 1: Calculating and determining the sample size to be 500 individuals. Step 2: the sample size for each city was determined based on the population size of the 14 cities in Liaoning Province. Step 3: Utilizing a systematic random method to identify the sampling communities. Step 4: A cluster sampling approach was applied to randomly select 5 workers from each community as the subjects of the survey. The sample size allocation is shown in Table 1. The survey was conducted through the distribution of paper questionnaires, with a total of 625 questionnaires distributed. After manually screening and excluding 80 invalid questionnaires (blank or with incorrect fields), 545 valid questionnaires were retained, resulting in a valid response rate of 87%, ensuring the generalizability and representativeness of the data. The sample selection chart is shown in Figure 1.

**Table 1:** Sample size allocation.

City	Amount of sample communities per city	Amount of samples per communities	Sample size
Shenyang	28	5	140
Dalian	17	5	85
Anshan	10	5	50
Chaoyang	9	5	45
Jinzhou	9	5	45
Huludao	8	5	40
Tieling	8	5	40
Yingkou	7	5	35
Dandong	6	5	30
Fushun	5	5	25
Fuxin	5	5	25
Liaoyang	5	5	25
Panjin	4	5	20
Benxi	4	5	20
Sum	125		625

### 3.2 Variable selection and measurement

#### 3.2.1 Occupational stress

This study adopted the Occupational Adaptability Scale for Employees (OASE) compiled by Wang to measure community workers’ occupational stress (Wang et al., 2016). The scale includes six factors, including performance appraisal, organizational collaboration, workload, vocational learning, emotional control and career development. It is scored using a 5-point Likert scale ranging from 1 for no pressure to 5 for great pressure. The higher the score, the greater the occupational pressure. The scale has been widely used in occupational psychology research and has good reliability and validity.

#### 3.2.2 Social support

In this study, the Social Support Rating Scale (SSRS) compiled by Xiao (1994) was adopted to measure community workers’ social support. The scale covers three dimensions including subjective support, objective support and utilization of support, with a total of 10 items. Subjective support consists of four items, which measures the emotional support experienced by individuals, including the care, understanding, respect and praise they receive. Objective support consists of three items, which measures the tangible support provided to individuals, including material assistance, economic support, and participation in social networks. The utilization of support has three items, which measures the utilization of various types of support by individuals. The subscale scores are totaled to yield an overall score with higher scores reflecting greater social support.

#### 3.2.3 Psychological health

In this study, SCL-90-R scale was used to measure the psychological health of community workers (Carrozzino et al., 2016). The scale has a total of 83 items, including negative emotions (e.g., fear, anxiety, and hostility) and other dimensions. It is scored using a 5-point Likert scale ranging from 1 (none) to 5 (severe), with higher total scores reflecting poorer psychological health. It is a commonly used tool to measure psychological health in occupational psychology research.

### 3.3 Statistical analysis

In this study, SPSS 13.0 software was used to conduct descriptive statistics (SPSS Inc, 2005), mean difference test, correlation analysis and adjustment effect analysis (Table 1).

## 4 Results

### 4.1 Occupational stress of community workers

To develop a preliminary understanding of community workers’ occupational stress, the occupational stress scores measured by the OASE and the total average scores are calculated and summarized in Table 2.

**Table 2:** Occupational adaptability scale for employee scores of community workers.

	Performance appraisal	Organization collaboration	Vocational learning	Emotional control	Workload	Career development	Overall average
Average score	3.03	2.80	2.81	2.05	3.68	3.28	2.93
Rank	3	5	4	6	1	2	

It can be seen in Table 2 that community workers generally feel considerable occupational stress, including stress due to their workload, career development opportunities, performance appraisal, vocational learning, organization collaboration, and emotional control in descending order.

### 4.2 Comparison of psychological health status of community workers under different stress conditions

To explore the relationship between occupational stress and psychological health status, the subjects were divided into two groups according to their occupational stress scores. Subjects whose average score was one standard deviation higher than the overall average were classified as a high stress group, with a total of 86 people. Subjects whose average score was one standard deviation below the overall average were classified as a low stress group with a total of 56 people. The SCL-90 scores for the high and low stress groups were calculated and compared using the student’s *t*-test and are shown in Table 3.

**Table 3:** Comparison of SCL-90-R scores between the high stress and low stress groups.

Factors	High stress group (*n* = 86)	Low stress group (*n* = 56)	*t*
Somatization	2.70 ± 0.99	1.42 ± 0.51	8.86^**^
Obsessive-compulsive symptoms	2.96 ± 0.95	1.44 ± 0.39	11.29^**^
Interpersonal sensitivity	2.57 ± 0.97	1.26 ± 0.30	9.75^**^
Depression	2.70 ± 0.93	1.31 ± 0.36	10.59^**^
Anxiety	2.53 ± 1.01	1.27 ± 0.29	9.13^**^
Hostility	2.60 ± 0.99	1.34 ± 0.40	8.98^**^
Fear	2.03 ± 0.96	1.14 ± 0.27	6.79^**^
Paranoid ideation	2.36 ± 0.96	1.20 ± 0.27	8.89^**^
Psychoticism	2.21 ± 0.89	1.22 ± 0.38	7.93^**^

^**^p < 0.01.

Table 3 shows that there are significant differences in the SCL-90-R factor scores between the high and low stress groups, and the scores of the high stress group are higher than those of the low stress group, indicating that occupational stress has a negative impact on community workers’ psychological health.

### 4.3 Comparison of the psychological health status of community workers with different levels of social support

To explore the relationship between social support and psychological health status, the subjects were grouped according to their social support scores, and those whose total score was one standard deviation higher than the overall average were classified as a high support group, with a total of 108 people. Subjects whose average score was one standard deviation below the overall average were classified as a low support group with a total of 87 people. The SCL-90-R scores were calculated for the two groups and compared using the student’s *t*-test and are shown in Table 4.

**Table 4:** Comparison of SCL-90-R scores between the high and low social support groups.

Factors	High social support group (*n* = 108)	Low social support group (*n* = 87)	*t*
Somatization	1.79 ± 0.63	2.41 ± 0.98	−5.35^**^
Obsessive-compulsive symptoms	1.83 ± 0.56	2.61 ± 0.98	−6.99^**^
Interpersonal sensitivity	1.50 ± 0.46	2.31 ± 1.02	−7.39^**^
Depression	1.56 ± 0.53	2.43 ± 0.98	−7.95^**^
Anxiety	1.52 ± 0.50	2.30 ± 1.01	−7.06^**^
Hostility	1.59 ± 0.52	2.37 ± 1.02	−6.87^**^
Fear	1.30 ± 0.42	1.85 ± 0.98	−5.18^**^
Paranoid ideation	1.41 ± 0.42	2.14 ± 0.95	−7.22^**^
Psychoticism	1.37 ± 0.41	2.06 ± 0.90	−7.12^**^

^**^p < 0.01.

Table 4 shows that there are significant differences in the SCL-90-R scores between the high and low social support groups, and the scores for all factors in the low support group are higher than those in the high support group. This indicates that social support has a positive impact on the psychological health status of community workers.

### 4.4 Correlations between community worker’s psychological health and occupational stress and social support

The correlation coefficients of psychological health factor scores with occupational stress and social support were calculated and are shown in Table 5.

**Table 5:** Correlations between SCL-90-R scores and organizational stress and social support.

	Performance appraisal	Organization collaboration	Vocational learning	Emotional control	Workload	Career development	Support score	Objective support	Subjective support	Support availability
Somatization	0.41^**^	0.40^**^	0.35^**^	0.28^**^	0.46^**^	0.36^**^	−0.30^**^	−0.21^**^	−0.26^**^	−0.21^**^
Obsessive-compulsive symptoms	0.47^**^	0.48^**^	0.42^**^	0.38^**^	0.52^**^	0.39^**^	−0.37^**^	−0.24^**^	−0.33^**^	−0.25^**^
Interpersonal sensitivity	0.45^**^	0.50^**^	0.45^**^	0.43^**^	0.45^**^	0.37^**^	−0.47^**^	−0.27^**^	−0.35^**^	−0.30^**^
Depression	0.46^**^	0.47^**^	0.42^**^	0.39^**^	0.49^**^	0.39^**^	−0.40^**^	−0.26^**^	−0.35^**^	−0.28^**^
Anxiety	0.44^**^	0.45^**^	0.40^**^	0.36^**^	0.46^**^	0.35^**^	−0.38^**^	−0.24^**^	−0.32^**^	−0.23^**^
Hostility	0.40^**^	0.42^**^	0.37^**^	0.36^**^	0.44^**^	0.34^**^	−0.36^**^	−0.24^**^	−0.25^**^	−0.22^**^
Fear	0.34^**^	0.37^**^	0.33^**^	0.35^**^	0.34^**^	0.27^**^	−0.32^**^	−0.24^**^	−0.25^**^	−0.22^**^
Paranoid ideation	0.45^**^	0.49^**^	0.47^**^	0.45^**^	0.42^**^	0.41^**^	−0.38^**^	−0.27^**^	−0.33^**^	−0.26^**^
Psychoticism	0.40^**^	0.43^**^	0.43^**^	0.46^**^	0.38^**^	0.35^**^	−0.40^**^	−0.27^**^	−0.34^**^	−0.28^**^

^**^p < 0.01.

Table 5 shows that all psychological health factors are positively correlated with the six occupational stress factors, and more than half of the correlation coefficients are greater than 0.4. All psychological health factors were negatively correlated with all dimensions of social support, and the correlation coefficients were slightly lower than those of psychological health and occupational stress. At the same time, subjective support has the most significant correlation with psychological health.

### 4.5 The moderating effect of social support on community workers’ occupational stress and psychological health

According to the requirements for moderating effects analysis, the test of the moderating effect of social support followed the following steps: (1) total occupational stress, social support and psychological health scores were converted to standard scores, (2) the interaction term “occupational stress × social support” was generated, (3) the total psychological health scores were used as the dependent variable for hierarchical regression analysis, and the main effect term and interaction term were introduced successively into the model. The significance of the moderating effect of social support was evaluated by examining the change in *R*^2^ as a result of the newly added variable (Δ*R*^2^) or the regression coefficient of the interaction term. As shown in Table 6, the explanatory value of the moderating effect reached significance (*p* < 0.01), indicating that social support has a moderating effect on the relationship between occupational stress and psychological health.

**Table 6:** Analysis of the moderating effects of social support.

	*R* ^2^	Δ*R*^2^	Δ*F*	*β*
Step 1	0.35	0.35	148.37^**^	
Occupational stress				0.47^**^
Social support				−0.25^**^
Step 2	0.39	0.04	29.11^**^	
Occupational stress				−0.45^**^
Social support				0.22^**^
Occupational stress × Social support				−0.15^**^

^**^p < 0.01.

**Figure 2:**
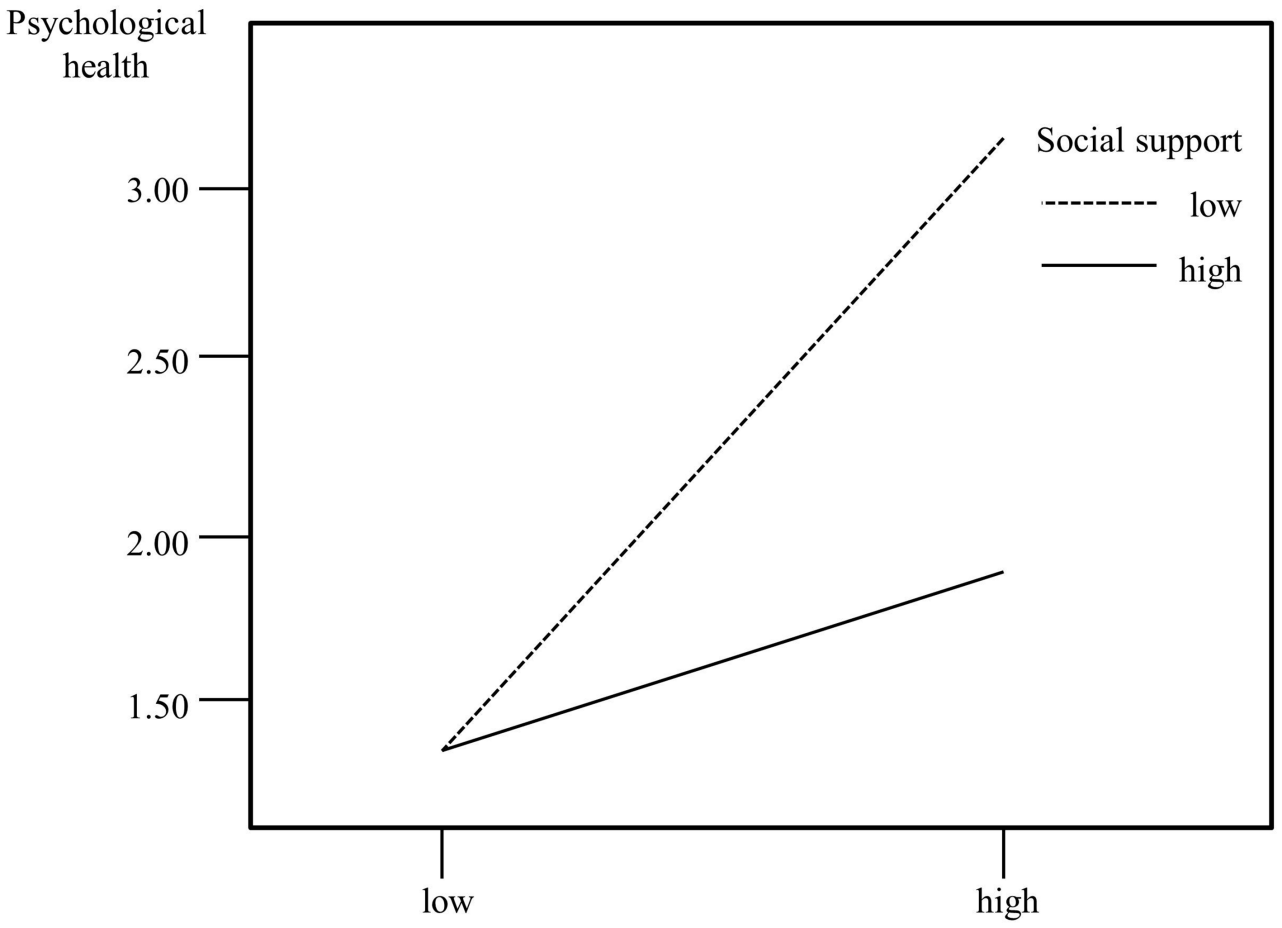
The relationship between social support, occupational stress and psychological health.

Table 6 also shows that occupational stress is closely related to psychological health. Occupational stress has a positive effect on the total psychological health scores, while social support has a negative effect. That is, greater occupational stress corresponds to higher psychological health scores and lower levels of health, while higher social support corresponds to lower overall psychological health scores and higher levels of health. When the variables occupational stress and social support entered the regression equation in the first step, the coefficient of determination *R*^2^ was 0.35, which increased to 0.39 when the interaction between the two variables was entered into the regression equation in the second step. The change in Δ*R*^2^ is 0.04, and the regression coefficient of the interaction is significant, indicating that social support regulates the relationship between occupational stress and psychological health. The influence of social support on occupational stress and psychological health is shown diagrammatically in Figure 2.

Figure 2 shows that under low occupational stress levels, there is little difference in the psychological health of the two groups with different levels of social support. By comparison, there is a large difference in the psychological health of the two groups with different levels of social support when occupational stress levels are high, and social support plays a moderating role in the impact of occupational stress on psychological health.

## 5 Discussion

### 5.1 The main stressors of community workers and their correlation with psychological health

This study indicates that the total occupational stress of community workers in this study is 2.93, reflecting relatively high stress levels. The top three stressors were workload, career development opportunities and performance appraisal, which was basically consistent with the research results of Ping et al. (2020). Workload is the biggest source of pressure for community workers. In addition to completing basic social management and public service tasks, they also spend considerable time on tedious tasks, such as organizing activities, attending conferences, accepting inspections by superior departments, and issuing certification materials, etc. According to statistics, most community workers work more than 10 h a day and overtime is widespread (Zhang and Liu, 2022). As China puts forward the strategy of “modernization of grassroots governance capacity,” the requirements for community workers’ knowledge and working capacity are increasing, and they are required to master innovative and effective working methods. Therefore, community workers must devote some time to undertaking various kinds of training. The second source of stress is career development, which reflects the incomes and social status of community workers. An individual’s motivation to work is frequently based on economic rationality and if his/her salary is insufficient or career development opportunities are limited, he/she is likely to experience pressure and a corresponding decline in his/her enthusiasm for work. In recent years, China has carried out systems innovation for the construction and development of communities, which has increased the community’s ability to mobilize social resources. The salaries of community workers have also increased somewhat, but a significant gap remains between their salaries and those of professionals such as doctors and lawyers. Moreover, opportunities for the promotion of community workers are limited, and the methods of promoting outstanding workers are vague, thereby increasing occupational pressure. The third source of stress is performance appraisal. Establishing valid and reliable performance appraisal systems with clearly defined expectations and feedback mechanisms is an important way to mobilize the enthusiasm of community workers, improve their work performance, and efficiency. However, the direct impacts of performance evaluation results on workers’ employment, bonuses, promotions, and other aspects, which are related to their development and honor, create considerable occupational stress.

Correlation analysis shows that community workers’ psychological health was significantly negatively correlated with occupational stress, that is, the greater the occupational stress, the lower their psychological health. Research by Karimi et al. (2014) supports this conclusion. Mullan (2014) believes that moderate occupational stress can eliminate feelings of emptiness and depression, enhance motivation, and help improve the efficiency of work and study. Conversely, excessive occupational stress results in distress, boredom, anxiety, and depression, and results in physiological and psychological disorders.

### 5.2 The main stressors of community workers and their correlation with psychological health

Previous studies have found that social support plays an important role in people’s physical and psychological health, and high levels of social support can provide protection for individuals experiencing stress and enhance their emotional well-being (Budge et al., 2013). This study further indicates that the psychological health of community workers is positively correlated with social support, and that higher levels of social support correspond to better psychological health. Of the types of support examined, the correlation between subjective support and psychological health was the greatest. Moderating effects analysis shows that social support modifies the relationship between occupational stress and community workers’ psychological health. In the current study, it was found that participants with high social support had better psychological health irrespective of the level of occupational stress. When experiencing occupational stress, community workers with high social support feel respected and cared for, have stronger self-esteem and self-efficacy, and receive sufficient material, information and spiritual support to cope with adverse situations and avoid negative emotions such as anxiety and depression. By comparison, community workers with low social support often feel undervalued, isolated, and helpless when facing stress, and find it difficult to vent their negative emotions, which damages their psychological health. Therefore, more attention should be paid to community workers with low social support.

## 6 Conclusion

Based on survey data from respondents in 14 cities in Liaoning Province, this study explored the relationship between occupational stress, social support and the psychological health of community workers. It was found that community workers face significant occupational stress, mainly due to their workload, career development opportunities, and performance appraisal. Community workers’ psychological health was significantly negatively correlated with occupational stress, and significantly positively correlated with social support with social support regulating the relationship between occupational stress and psychological health.

Based on these findings and China’s policy of “modernization of grassroots governance capacity,” the following suggestions are proposed to improve community workers’ health and the community governance system:

First, all sectors of society should recognize the important role of community workers and their contribution to society, understand their work environment, and pay attention to their health status.

Second, reduce communities’ burden, establish and implement the community affairs list system, transfer matters that do not belong to community departments to other departments, eliminate repetitive and inefficient work practices, optimize work processes, and establish a work model of solidarity and cooperation.

Third, establish an effective community support system, including increasing the number of community workers and their salaries, broadening their career promotion opportunities, and establishing a sound reward and performance appraisal system, to promote their professional identity and enthusiasm for work.

Fourth, create a positive working environment for community workers, implement a democratic internal management model, create an atmosphere of tolerance, cooperation and mutual assistance, and provide psychological counseling for community workers through recreational activities and symposiums to address negative work-relation emotions.

The implications of this study are multifaceted: firstly, at the theoretical level, this study constructs a structural equation model that delineates the relationships among community workers’ occupational stress, social support, and psychological health. Secondly, at the practical level, the model has been empirically tested against a substantial dataset, providing a solid basis for practical application. Thirdly, at the societal level, this study offers a foundation for community development policies, thereby contributing to the improvement of workers’ health and the promotion of sustainable development in communities both in China and globally.

The limitations of this study are noted as follows: the research was conducted in Liaoning Province, which, while being representative for the purposes of this study, may limit the generalizability of the findings. Future research could expand the geographical scope and sample size to encompass a broader spectrum of communities, and comparative analyses across different regions could be conducted to enhance the applicability and robustness of the results.

## Data Availability

The original contributions presented in the study are included in the article/supplementary material, further inquiries can be directed to the corresponding author.
